# Gait and Balance Changes with Investigational Peripheral Nerve Cell Therapy during Deep Brain Stimulation in People with Parkinson’s Disease

**DOI:** 10.3390/brainsci11040500

**Published:** 2021-04-15

**Authors:** Geetanjali Gera, Zain Guduru, Tritia Yamasaki, Julie A. Gurwell, Monica J. Chau, Anna Krotinger, Frederick A. Schmitt, John T. Slevin, Greg A. Gerhardt, Craig van Horne, Jorge E. Quintero

**Affiliations:** 1 Department of Physical Therapy, College of Health Sciences, University of Kentucky, 204L 900 South Limestone Street, Lexington, KY 40536, USA; 2 Brain Restoration Center, University of Kentucky, Lexington, KY 40536, USA; zain.guduru@uky.edu (Z.G.); tyamasaki@uky.edu (T.Y.); jagur@email.uky.edu (J.A.G.); monica.chau@uky.edu (M.J.C.); fascom@uky.edu (F.A.S.); jslevin@email.uky.edu (J.T.S.); gregg@uky.edu (G.A.G.); craigvanhorne@uky.edu (C.v.H.); george.quintero@uky.edu (J.E.Q.); 3 Neurology, University of Kentucky, Lexington, KY 40536, USA; 4 Neuroscience, University of Kentucky, Lexington, KY 40536, USA; 5 Veterans Affairs Medical Center, Lexington, KY 40502, USA; 6 Neurosurgery, University of Kentucky, Lexington, KY 40536, USA; 7 Department of Neuroscience, Wesleyan University, Middletown, CT 06459, USA; annakrotinger@gmail.com

**Keywords:** deep brain stimulation, gait, balance, cell therapy, Parkinson’s disease

## Abstract

Background: The efficacy of deep brain stimulation (DBS) and dopaminergic therapy is known to decrease over time. Hence, a new investigational approach combines implanting autologous injury-activated peripheral nerve grafts (APNG) at the time of bilateral DBS surgery to the globus pallidus interna. Objectives: In a study where APNG was unilaterally implanted into the substantia nigra, we explored the effects on clinical gait and balance assessments over two years in 14 individuals with Parkinson’s disease. Methods: Computerized gait and balance evaluations were performed without medication, and stimulation was in the off state for at least 12 h to best assess the role of APNG implantation alone. We hypothesized that APNG might improve gait and balance deficits associated with PD. Results: While people with a degenerative movement disorder typically worsen with time, none of the gait parameters significantly changed across visits in this 24 month study. The postural stability item in the UPDRS did not worsen from baseline to the 24-month follow-up. However, we measured gait and balance improvements in the two most affected individuals, who had moderate PD. In these two individuals, we observed an increase in gait velocity and step length that persisted over 6 and 24 months. Conclusions: Participants did not show worsening of gait and balance performance in the off therapy state two years after surgery, while the two most severely affected participants showed improved performance. Further studies may better address the long-term maintanenace of these results.

## 1. Introduction

Parkinson’s disease (PD) is a neurodegenerative disease characterized in part by a progressive loss of dopamine due to neurodegeneration in the substantia nigra [1]. This progressive loss of dopamine, with up to 10% of the remaining dopaminergic cells dying each year, is a major cause of disability in those with PD, particularly related to gait and balance disturbances [2,3]. Gait and balance disturbances are one of the most important causes of diminished quality of life, morbidity, and mortality in people with PD [4]. Reduced gait speed and step length and impaired rhythmicity are all components of gait that worsen as the disease progresses [5]. Balance performance in people with PD is measured by postural control, rigidity, the center of foot pressure (CoP) displacement, velocity, and frequency [6]. Both gait and balance disturbances lead to increased falls, difficulty navigating a home or community environment, and increased overall morbidity [4]. Thus, interventions targeting the causes of disability in those with PD can reduce mortality and improve independence and quality of life in this population [4]. While there is no cure for PD, levodopa is the gold standard medication for managing PD symptoms but does not alter the pathophysiological trajectory [4].

Deep brain stimulation (DBS) is a therapy for PD in which electrodes are surgically implanted in the subthalamic nucleus (STN) or globus pallidus interna (GPi), areas, which are responsible for motor control. However, the efficacy of the combination of DBS and dopaminergic therapy is known to decrease over time. Some studies suggest this may be because traditional therapies are not targeting the specific cause of the disease. Thus, the patients still experience disease progression over time [7].

The decreased efficacy of DBS and dopaminergic therapies over time has brought forth a new, experimental combined approach of implanting autologous injury-activated peripheral nerve grafts (APNG) at the time of DBS surgery. We have preliminarily found that this approach can be safe and feasible [8,9]. In our approach, the participant’s own peripheral (sural) nerve tissue is implanted into the substantia nigra as investigational cell therapy. Our strategy is to support the existing neurons in the substantia nigra and prevent degeneration. We implant sural nerve tissue that had been transected to convert the associated Schwann cells into a reparative phenotype [10,11,12,13]. After peripheral nerve transection or injury, Schwann cells undergo a unique cellular reprogramming that transdifferentiates the cell from a myelinating phenotype into a reparative phenotype [10,11,13,14]. The cells then upregulate and release a whole host of neurotrophic and cell survival factors, including glial-derived neurotrophic factor (GDNF) [15], nerve growth factor (NGF) [16,17], brain-derived neurotrophic factor (BDNF) [18], and nuclear factor erythroid 2-related factor (Nrf2) in animal models. Our RNA Seq analyses support that the human sural nerve tissues that we implanted show similarities to the repair phenotype in animal models [19]. Our strategy is to use APNG to implant a living repair system with growth factors to reduce cell death of dopamine-producing neurons and aid in the long-term maintenance of motor function, including gait and balance.

Here we report the effects of APNG on various balance and gait measures, including step length, velocity, and cadence over two years. As part of a post hoc analysis, we hypothesized that APNG might improve gait and balance deficits associated with PD. If APNG were subsequently shown to be efficacious, it could alter the management of PD, allowing those with this disease to retain their mobility for a better quality of life.

## 2. Materials and Methods

**Figure 1:**
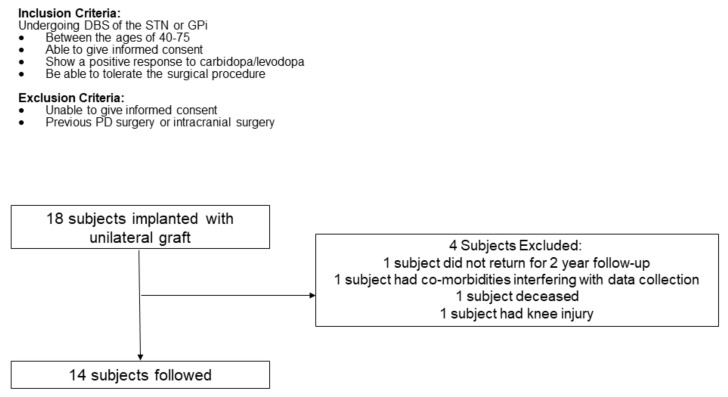
Participants inclusion flowchart.

Study design. This report presents a subpopulation of participants who completed gait and balance assessments from a 24-month, investigator-initiated, open-label, single-center, Phase I trial designed to assess the safety and feasibility of the direct delivery of peripheral nerve tissue APNG into the substantia nigra (NCT02369003). Gait and balance were exploratory outcomes of the trial and are reported in detail here separately from the rest of the study thus adverse events are not reported. However, as reported in our earlier work, the most common adverse events related to the study intervention were paresthesia, numbness, and pain of the ankle and foot immediately following the sural nerve biopsy [9]. Eighteen participants were implanted with a unilateral graft; however, 14 participants were included in these analyses (Figure 1). Participants were evaluated at baseline, before surgery, and every 6 months for 24 months. (*N* = 14; 61.9 ± 8.3 y; 10 M/4 F; OFF medication UPDRS III = 37.6 ± 11.8) (Figure 1).

Participant selection. As previously described [8,9], patients, who were good candidates for DBS for PD were offered the opportunity to enroll if they met the inclusion/exclusion criteria. The eligibility criteria used for the screening phase are presented in Figure 1. The University of Kentucky institutional review board (IRB) approved the study, and all participants provided informed consent.

### 2.1. Surgical implantation

In the current trial, APNG was implanted after the bilateral DBS electrodes had been placed in the GPi (STN for one participant), tested, secured to the skull, and connected to the lead extensions. The specific details for graft injury conditioning, graft harvest and implantation have been described in detail previously [8,9]. Grafts were implanted unilaterally into the substantia nigra contralateral to the most affected side based on the lateralized Unified Parkinson’s Disease Rating Scale (UPDRS) part III scores. After DBS electrode and tissue implantation procedures, a postoperative MRI was used to aid in confirming electrode placement and APNG implant. Participants underwent routine postoperative visits, programming visits, and postoperative clinical study visits.

### 2.2. Clinical Testing

Participants stopped all the dopaminergic medications, and DBS stimulation was turned off at least 12 h before UPDRS III testing and gait/balance testing. UPDRS Part III is the motor examination component of the assessment that includes an evaluation of gait. UPDRS item 30 (postural stability) was used to access balance. The UPDRS item 30 (postural stability) scale ranges from 0 to 4, with 0 representing normal/no problems and 4 representing a severe loss of balance. Hoehn and Yahr’s (HY) score was also assessed, based on the severity of the disease before undergoing the procedure and every 6 months after the procedure for 2 years. The HY scale is used to describe PD progression and includes stages 1 through 5, with 1 representing minimal, unilateral symptoms and 5 representing confinement to a bed or wheelchair unless aided.

### 2.3. Gait Data Collection

Each participant performed three walking trials at their self-selected speed across the 24 foot GAITrite^®^ walkway (CIR Systems, Franklin, NJ, USA). We present the data for the participants in the OFF state (off levodopa at baseline for at least 12 h) at the time of enrollment and off levodopa/off DBS at least 12 h at 6 months and 24 months post-surgery. OFF state (OFF levodopa and OFF DBS) gait data were tested to determine the change in gait performance, as part of the evaluation of the underlying disease progression, in the absence of a standard of care therapy. Gait data were analyzed using the GAITrite software. The data collected were uploaded into an excel file for data summaries.

### 2.4. Outcome Variables

The gait variables measured and analyzed using the GAITrite software included spatial measures (step length and velocity) and a temporal measure (cadence). Cadence is defined as the total number of steps taken in a minute. Step length is the distance between the point of initial contact of one foot and the point of initial contact of the opposite foot. We used a within-subjects repeated measures ANOVA design to investigate if the gait metrics changed across visits (SPSS 26). We set the threshold for a significant difference to *p* < 0.05. When there was a significant within-subjects effect, we examined the pair-wise comparisons to determine the source of significance (no adjustments were performed for the pair-wise comparison). The clinical scales evaluated were UPDRS III total (overall score), UPDRS item 29 (gait), UPDRS item 30 (postural stability) and Hoehn and Yahr. Analysis was performed on 14 individuals, as shown in Figure 1.

## 3. Results

### 3.1. Changes in Clinical Scales 6 and 24 Months after APNG Implantation

**Figure 2:**
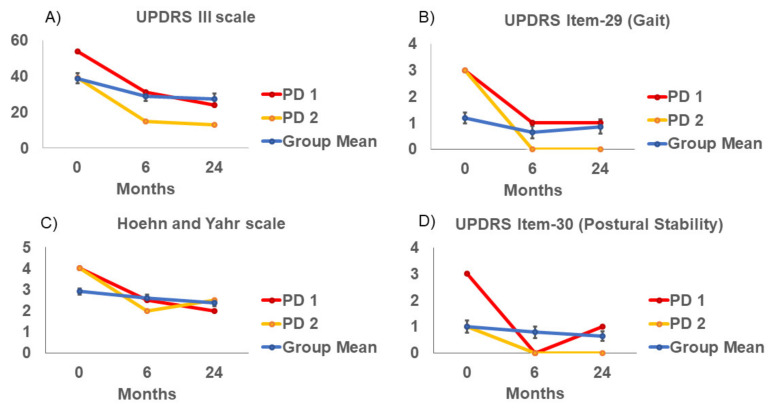
(**A**) UPDRS III (**B**) UPDRS III Item-29 (Gait) (**C**) Hoehn and Yahr and (**D**) UPDRS III Item-30 (Postural Stability) clinical scales at baseline (0 month), 6 and 24 months post-surgery. Group means (SE), and example subjects: Parkinson’s disease (PD) subject 1 (UPDRS = 54), and PD subject 2 (UPDRS = 39).

We report the effect of APNG implantation, if any, on clinical scales (UPDRS III, UPDRS, item 29 (gait), HY, and UPDRS, item 30 (postural stability), across 0, 6 and 24 months among this cohort (Table 1, Figure 2). Evaluations were performed off medication and off stimulation for at least 12 h to best assess the potential effect of APNG. UPDRS Part III mean scores were lower at 6 and 24 months than at baseline. HY scale also showed a lower value at 24 months than at baseline. UPDRS–item 30 (postural stability) mean scores did not worsen from baseline to 6 and 24 months. Gait component of the UPDRS, item 29 (gait), approached significant difference (*p* = 0.051).

### 3.2. Gait Measures for the Cohort

**Figure 3:**
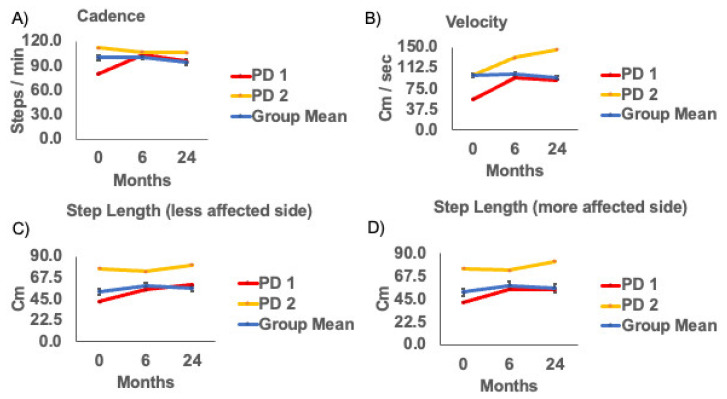
**(A**) Cadence (**B**) Velocity (**C**) Step length (less) and (**D**) Step length (more) affected sides at baseline(0 month), 6 and 24 months post-surgery. Group means (SE), and example subjects: Parkinson’s disease (PD) subject 1 (UPDRS = 54), and PD subject 2 (UPDRS = 39).

None of the changes in gaitmeasureswere statistically significant across visits. Thus, overall, there was no visit effect of the APNG for gait measures from zero to 6 months or 24 months, suggesting no statistically significant decrement or improvement in gait parameters longitudinally in this cohort (Table 2, Figure 3). However, gait velocity (*p* = 0.07) and step length for the more affected side (*p* = 0.08) approached significance.

### 3.3. Gait Measures for Subjects with Deficits in Gait and Overall Disease Severity

The cohort in our study was heterogeneous for disease severity and gait/balance deficits. Moreover, our participants were less severely affected compared to participants tested in previous studies. In our cohort, there were two participants, who had UPDRS scores > 37, classified as moderate disease severity and a gait deficit > 2, also considered a moderate deficit. Unlike the overall group, these two participants showed a reduction in total UPDRS III, UPDRS item 29, HY scores, and UPDRS item 30 (Figure 2). These two participants also showed an increase in gait velocity and step length at 6 months and 24 months (Figure 3).

## 4. Discussion

We explored the effects of unilateral substantia nigra APNG on gait and balance deficits. We focused solely on the role of APNG and not on the contribution of DBS or medication by specifically examining participants at times when they were off medication and off stimulation. While we cannot rule out that stimulation and/or medication may have had prolonged effects beyond the washout period, the length of the washout period for assessments was consistent before and after surgery. We quantified the effects of APNG on gait and balance for up to 2 years. Gait and balance components did not deteriorate in our cohort over 6 and 24 months postoperatively in DBS OFF and medication OFF state. As PD is a progressive neurodegenerative disease where with time, symptoms would worsen, this suggests a possible ameliorative effect. The results reported here bolster our approach providing this investigational cell therapy while still being able to deliver the standard of care for symptomatic relief using DBS.

DBS studies have revealed improvements in gait measures with an increase in step length, gait velocity, and reduced double stance time [4]. Temporal measures of gait, such as cadence, may not change or decrease with DBS [6,20,21,22,23,24,25,26,27,28,29,30,31]. Studies that measured changes in gait parameters before and after DBS electrode implants examined participants, who were more affected than in this report, with an average UPDRS of 50 and average HY of 3.7 in the OFF-medication state at baseline [20,32]. Our participants’ PD severity was rated less symptomatic than these studies (Table 1). Gait deficits were also more prominent in these published studies, with a gait velocity of approximately 0.74 m/s at baseline compared to our cohort with gait velocity at 0.83 m/s. Thus, individuals included in our study were less severely affected and may have had a smaller range in which to display improvements; however, when observing the results through the lens of disease progression, notably, we did not find worsening in gait measures in the OFF state two years after baseline. We will continue to follow participants yearly and re-evaluate their gait performance. By evaluating over a longer time frame, APNG intervention could be shown to slow the progression of the disease even in the less severely affected individuals. With regard to time frame, we note that Defebreve et al. assessed individuals at three months post-surgery, whereas ours extended to 24 months [32].

We further investigated two individuals in our cohort who had moderate PD. In both of these individuals that were most affected by PD, we observed an increase in gait velocity as well as an increase in step length that persisted over 6 and 24 months. There was a reduction in the gait component of the UPDRS score as well as the total UPDRS-III score at both 6 and 24 months. The improved score continued over 6 and 24 months compared to baseline for the postural stability component of the UPDRS score. Changes in OFF state scores were not accounted for by interval medication changes for these participants. In line with the usual therapeutic response to DBS, medications were reduced in both participants. Participant 1 remained on stable doses of rasagiline and had reduced doses of both ropinirole (60% reduction–24 h washout) and levodopa (75% reduction) by the 24 month period. Participant 2 was on a combination of levodopa, rasagiline and rotigotine patch at baseline and by 24 months was on levodopa only (<50% of his baseline dose). Neither participant was on extended-release formulations of levodopa medication. Additionally, testing occurred after a washout period (24 h–ropinirole; 48 h–rotigotine patch) in accordance with the half-life of the medication.

Importantly, Allert et al. reported a 35% increase in the gait velocity in 3 months in the off state [20]. In our defined OFF state (OFF stimulation, OFF levodopa), Participant 1 (PD1) experienced a 70% increase in gait velocity at 6-month and a 64% increase at 24-month from baseline. Participant 2 (PD2) experienced a 32% increase in gait velocity at 6-month and a 45% increase at 24-month from baseline. In the absence of DBS stimulation and medication treatment, these individuals walked faster with longer step lengths than their baseline gait measures. Although at this stage, this interpretation remains speculative, and further examination of the effects on gait performance will need to be studied, including in participants receiving bilateral APNG to the substantia nigra.

Our observation of changes in more severely affected participantscouldemerge during subsequent measures in the less severely affected individuals as their disease progresses. Future studies must also target more severely affected individuals to determine if our finding in the two moderately affected participants is more than anecdotal.

### Limitations

The small sample size and limited heterogeneity may limit projecting the findings to the general population of patients with PD. In addition, because of the early stage of this trial, there was neither a DBS-only control group nor a medication-only control group included in this small exploratory study. The absence of a comparison group limited our ability to compare natural declines in gait and balance performance over time among individuals and limited our ability to draw generalizable conclusions.

## 5. Conclusions

Here we show that participants with PD, who received a unilateral APNG to the substantia nigra, did not show worsening of gait and balance performance two years after surgery. Further studies may better address the efficacy and stability of these results.

## Figures and Tables

**Table 1:** Means, (95% Confidence Interval) of clinical scales at baseline (0 month), 6 and 24 months post-surgery.

	0 months	6 months	24 months	F-Score, *P*, η_p_^2^
UPDRS III	37.6 (30.8−44.5)	29.1 (23.4−34.7) ^a^	27.4 (21.3−33.6) ^b^	F_2, 26_ = 9.43, *p* < 0.05, 0.42
UPDRS III (Gait)	1.3 (0.7−1.9)	0.6 (0.32−1.0)	0.9 (0.4−1.3)	F_2, 26_ = 3.34, *p* = 0.051, 0.21
Hoehn and Yahr	3.0 (2.6−3.4)	2.6 (2.4−2.8)	2.4 (2.1−2.6) ^b^	F_2, 26_ = 4.59, *p* < 0.05, 0.26
UPDRS III (Postural Stability)	1.0 (0.5−1.5)	0.8 (0.3−1.2)	0.6 (0.2−1.1)	F_2, 26_ = 0.815, *p* = 0.45, 0.06

^a^ Significant difference (*p* < 0.01) between 0 and 6 months. ^b^ Significant difference (*p* < 0.01) between 0 and 24 months. ηp^2^ is partial eta squared = effect size.

**Table 2:** Means, (95% Confidence Interval) of gait parameters at baseline (0 month), 6 and 24 months post-surgery.

	0 months	6 months	24 months	F-Score, *P*, η_p_^2^
Cadence	97.71 (90.84−104.59)	101.19 (94.82−107.55)	94.04 (85.22−102.86)	F_2, 26_= 2.05, *p* = 0.15, η_p_^2^= 0.14
Velocity	82.58 (66.91−98.25)	99.14 (84.32−113.96)	89.72 (71.65−107.79)	F_2, 26_= 2.92, *p* = 0.07, η_p_^2^ = 0.18
Step Length(less affected side)	52.55 (43.20−61.90)	59.03 (52.11−65.95)	56.55 (48.54−64.55)	F_2, 26_= 2.54, *p* = 0.1, η_p_^2^ = 0.16
Step Length(more affected side)	51.43 (41.97−60.89)	58.19 (50.72−65.66)	55.86 (46.80−64.91)	F_2, 26_= 2.83, *p* = 0.08, η_p_^2^ = 0.18

η_p_^2^ is partial eta squared = effect size.

## Data Availability

The data are not publicly available because of privacy concerns.
